# Molecular Mechanisms of Root Exudate-Mediated Remediation in Soils Co-Contaminated with Heavy Metals and Polycyclic Aromatic Hydrocarbons

**DOI:** 10.3390/toxics13121044

**Published:** 2025-12-02

**Authors:** Lingyun Sun, Jinling Mo, Zhenjiang Wang, Sen Lin, Dan Wang, Zhiyi Li, Yuan Wang, Jianan Wu, Wuyan Guo, Jiehua Chen, Zhipeng Wu, Lian Chen

**Affiliations:** 1 Sericultural & Agri-Food Research Institute, Guangdong Academy of Agricultural Sciences, Guangzhou 510610, China; 2 School of Breeding and Multiplication (Sanya Institute Breeding and Multiplication), Hainan University, Sanya 572025, China; 3 Key Laboratory of Urban Agriculture in South China, Ministry of Agriculture and Rural Affairs, Guangzhou 510610, China

**Keywords:** heavy metals, polycyclic aromatic hydrocarbons, root exudates, rhizosphere microorganisms, phytoremediation

## Abstract

Soil co-contamination with heavy metals (HMs) and polycyclic aromatic hydrocarbons (PAHs) represents a widespread and challenging environmental issue that is difficult to address using conventional remediation methods. This review systematically examines the molecular mechanisms by which plant root exudates mediate the remediation of co-contaminated soils through synergistic interactions with rhizosphere microorganisms. We detail how plants dynamically adjust the composition and secretion of root exudates—such as organic acids, amino acids, sugars, and secondary metabolites—in response to combined HM-PAH stress. These exudates play multifaceted roles in remediation, including chelating HMs, enhancing PAH solubility and bioavailability, and acting as chemoattractants and metabolic substrates for rhizosphere microbes. In return, the recruited microbial communities contribute to pollutant detoxification through various mechanisms, such as biosurfactant production, enzymatic degradation, and improved plant nutrient acquisition. This reciprocal interaction forms a synergistic plant-microbe feedback loop that effectively mitigates combined contamination stress. By integrating evidence from diverse plant–soil systems, this review provides a comprehensive mechanistic framework for understanding root exudate-microbe interactions, offering critical insights for developing enhanced phytoremediation strategies to address complex environmental pollution.

## 1. Introduction

Approximately 95% of global food production is derived from terrestrial ecosystems, within which soil plays an indispensable role in the storage, transformation, and recycling of nutrients vital for sustaining human life. As the fundamental bedrock underpinning food security, ecological balance, and human health, healthy soil is essential for achieving sustainable development goals and ensuring environmental security. However, soil health is increasingly threatened by persistent contaminants. Among these, heavy metals (HMs)—such as arsenic (As), cadmium (Cd), chromium (Cr), lead (Pb), and mercury (Hg)—represent a prominent class of inorganic pollutants. Characterized by high density (typically >5 g/cm^3^), toxicity, and resistance to natural degradation, HMs originate from both natural processes like rock weathering and widespread anthropogenic activities including mining, smelting, industrial discharge, and agricultural practices [1].

Alongside HMs, polycyclic aromatic hydrocarbons (PAHs) constitute a major group of persistent organic pollutants, primarily formed from the incomplete combustion of fossil fuels and biomass. Their hydrophobic nature, persistence, and potential for bioaccumulation make them a significant environmental concern, with compounds like benzo[a]pyrene (BaP) recognized as highly carcinogenic priority pollutants by the US EPA [2]. According to the United Nations Global Soil Pollution Assessment Report, nearly 500 million hectares of soils worldwide are contaminated, with HMs and PAHs being widespread contributors. While HMs and PAHs differ markedly in their physicochemical properties, their prolonged co-occurrence leads to complex interactions in soil. This results in combined stress effects that are more variable and unpredictable than those from single contaminants, thereby intensifying ecological risks and elevating the threat of irreversible damage to soil structure and function [3,4]. Consequently, the effective remediation of co-contaminated soils has become a pressing global priority.

Conventional physicochemical remediation methods often prove inadequate for such complex scenarios, as they can be cost-prohibitive, disruptive to soil structure, and may generate secondary pollution [5]. In contrast, phytoremediation—particularly approaches that leverage the synergistic plant-microbe interactions within the rhizosphere—offers a more sustainable and eco-compatible alternative. The rhizosphere, defined as the narrow soil zone directly influenced by root activity, serves as a dynamic hotspot for biogeochemical processes. Here, plants actively release a diverse and complex mixture of root exudates, including low-molecular-weight organic acids, amino acids, sugars, and secondary metabolites, which collectively reshape the physical structure, chemical equilibrium, and biological functionality of the surrounding soil [6].

These root exudates are not merely passive metabolites; they function as active mediators of plant stress response and key facilitators of in situ remediation. They operate through several interconnected mechanisms: primarily by altering pollutant bioavailability through chelation, solubilization, or precipitation [7]; serving as chemoattractants and metabolic substrates to regulate microbial community composition and activity [6]; and optimizing the rhizosphere microenvironment to favor detoxification processes [8]. This sophisticated chemical dialogue establishes a self-reinforcing plant-microbe feedback loop that is fundamental to the phytoremediation of co-contaminated soils [9]. However, despite these recognized functions, a systematic and mechanistic understanding of how root exudates orchestrate the remediation of combined HM-PAH pollution remains fragmented. Critical knowledge gaps persist regarding the dynamic regulation of exudate profiles under dual stress, the precise molecular mechanisms by which they simultaneously mediate the fate of both inorganic and organic contaminants, and their role in assembling a synergistic, pollutant-degrading microbiome. Therefore, this review aims to synthesize current knowledge and elucidate the molecular mechanisms through which root exudates mediate the remediation of soils co-contaminated with HMs and PAHs, with a specific focus on the dynamic interplay between root exudates and rhizosphere microorganisms.

## 2. Research Progress Domestically and Internationally

### 2.1. Comprehensive Analysis of Root Exudate Composition and Rhizosphere Microorganism Interactions

Root exudates are complex mixtures of compounds actively secreted by plant roots into the rhizosphere, accounting for approximately 5–30% of total photosynthetically fixed carbon allocated to the rhizospheric compartment [10]. The release of root exudates functions as an adaptive strategy that mediates chemical, physical, and biological interactions between plants and complex soils. This process contributes to root protection under adverse environmental conditions while simultaneously enhancing plant–microbe interactions within the soil ecosystem. The composition and types of root exudates are shaped not only by intrinsic factors—such as plant variety [11] and growth stage [12]—but also by extrinsic variables, including soil physicochemical properties [13], climatic conditions [14], microbial communities [15], and nutritional status [16]. Root exudates comprise a wide array of compounds, broadly categorized into low-molecular-weight compounds (e.g., organic acids, amino acids, sugars, and secondary metabolites) and high-molecular-weight compounds (e.g., proteins and polysaccharides) [6]. While these exudates largely serve as carbon and nitrogen sources for rhizosphere microorganisms, specific compounds (particularly some secondary metabolites and signal molecules) play pivotal roles in regulating plant-microbe interactions [17]. A comprehensive investigation of root exudate composition, the regulatory mechanisms governing their secretion under various environmental and biological factors, and the associated methodologies is essential for improving plant adaptability to combined stresses, alleviating rhizospheric nutrient competition, and optimizing the structure–function relationships of rhizosphere microbial communities.

The composition of plant root exudates undergoes dynamic changes in response to environmental stresses, functioning as a pivotal adaptive strategy [18]. Under heavy metal stress (e.g., Cd, As), plants increase the secretion of organic acids (e.g., citric acid, malic acid) and phenolic compounds (e.g., flavonoids) into the rhizosphere, which can chelate metal ions and reduce their bioavailability, Inside the root cells, heavy metal stress also induces the synthesis of phytochelatins (PCs) and metallothioneins (MTs), which play essential roles in intracellular metal sequestration and detoxification [19,20]. Under organic pollution such as PAHs, plants secrete oxidases (e.g., laccase, peroxidase) and other metabolites that can enhance the solubilization and degradation of hydrophobic pollutants, thereby facilitating microbial degradation [21]. Under saline-alkali stress, root exudates exhibit significantly elevated levels of compatible solutes (proline, betaine) and organic acids (e.g., malate, succinic acid), which mitigate ion toxicity through osmoregulation and rhizosphere acidification [22]. Under drought conditions, plants secrete polysaccharides to enhance rhizosphere aggregate structure and release abscisic acid (ABA), thereby promoting microbial drought resistance [23]. Under nutrient deficiency, plant secretion mechanisms exhibit functional specificity tailored to alleviate particular nutrient limitations. For example, phosphatase secretion is upregulated under phosphorus deficiency to mobilize insoluble phosphorus, whereas siderophore secretion increases under iron limitation to facilitate efficient chelation of Fe^3+^ ions [24,25]. Notably, combined stresses from HMs and PAHs can exert synergistic or antagonistic effects on metabolic component secretion. For example, the simultaneous secretion of organic acids and biosurfactants can concurrently immobilize heavy metals while enhancing the solubilization of PAHs. However, elevated heavy metal concentrations may suppress the secretion of oxidases induced by PAHs [26]. These differences suggest that the dynamic modulation of root exudates represents not only a fundamental mechanism for plant stress adaptation but also a critical focus area for developing ecological restoration strategies centered on rhizosphere regulation.

Rhizosphere microorganisms, encompassing all microbial communities inhabiting the soil region surrounding plant roots, form a complex and dynamic ecosystem. This environment harbors extraordinary microbial diversity, comprising primarily bacteria, fungi, actinomycetes, protozoa, algae, and archaea, with bacteria and fungi typically constituting the dominant groups [27]. The composition and abundance of rhizosphere microorganisms are influenced by multiple factors, including plant species, growth stage, root exudates (e.g., sugars, amino acids, and organic acids), soil properties (such as type, pH, moisture, temperature), and nutrient availability [28]. A complex interplay exists between rhizosphere microorganisms and plants, encompassing mutualistic and competitive to parasitic relationships [29]. Certain rhizosphere microorganisms promote plant growth by facilitating nitrogen fixation, solubilizing phosphorus and potassium, secretion of plant hormones (e.g., auxins and cytokinins), and siderophore production. These mechanisms enhance nutrient acquisition and stress resilience in plants; conversely, other microbial inhabitants function as pathogens, causing various plant diseases [30]. As indispensable components of soil ecosystems, rhizosphere microorganisms play a pivotal role in maintaining soil health, regulating nutrient cycling, and modulating plant growth [27].

Soil serves as a vital habitat and optimal natural medium for microorganisms, wherein root exudates modulate the structure and function of rhizosphere microbial communities through diverse mechanisms. Evidence indicates that stress-induced shifts in root exudate profiles facilitate the recruitment of beneficial microbiota, thereby enhancing plant fitness and stress resilience [31]. Specifically, under varying environmental conditions, plants secrete diverse root exudate profiles that modulate rhizosphere microbial communities and foster a growth-favoring environment [32]. Root exudates exert dual effects on rhizosphere microbial communities by promoting the proliferation of beneficial microorganisms while concurrently suppressing the activity of pathogenic bacteria [33]. Zhou X et al. demonstrated that alterations in tomato root exudates, triggered by potato-derived chemical signals promote disease-suppressive rhizosphere microbiome establishment [34]. Furthermore, Seitz et al. demonstrated that root exudates mobilize specific ecosystem services from soil microbiomes—most notably enhanced disease resistance—through chemical signaling mechanisms [35]. These findings demonstrate that plants can enhance their stress adaptability and resilience by modulating root exudates, thereby optimizing the structure and functionality of rhizosphere microbial communities.

### 2.2. Effects of HMs-PAHs on Root Exudates Under Stress Conditions

#### 2.2.1. Impact of Heavy Metal Stress on Root Exudates

Heavy metal stress significantly disrupts key physiological and biochemical processes in plants, typically manifesting as growth suppression characterized by reduced plant height, decreased leaf area, and altered soluble protein content [36,37]. Heavy metal stress typically leads to a decline in chlorophyll content, thereby impairing photosynthetic function. For instance, in species like ginseng, mulberry, and cattail, heavy metals (e.g., Pb, Cd, Cu) inhibit chlorophyll synthesis, directly reducing photosynthetic efficiency [37,38,39]. Heavy metals compromise photosynthesis at the molecular level by targeting both the Calvin cycle (inactivating enzymes like Rubisco) and the Photosystem II (PSII) electron transport chain, leading to a significant decline in photosynthetic performance [40]. Heavy metal stress also triggers the overproduction of reactive oxygen species (ROS), thereby inducing oxidative damage that further exacerbates cell membrane injury. This is directly evidenced by a marked increase in the content of malondialdehyde (MDA) [41]. Plants employ a coordinated antioxidant defense system to counteract oxidative stress. The enzymatic components (e.g., SOD, POD, CAT) frequently show a biphasic activity pattern: they are upregulated under mild stress to scavenge ROS but become markedly suppressed under severe metal toxicity due to irreversible redox imbalance. Simultaneously, plants deploy a non-enzymatic defense system, which involves the synthesis and accumulation of protective metabolites. This includes increased production of phenolic compounds (including flavonoids and phenylpropanoids), glutathione, and proline, which can function as effective metal chelators and ROS scavengers [42,43,44].

As a defense strategy, plants dynamically reshape the composition and secretion of root exudates under heavy metal (HM) stress, most commonly increasing the release of low-molecular-weight organic acids (LMWOAs), amino acids, sugars, and secondary metabolites in a manner dependent on exposure time and HM concentration [45]. For instance, maize under high Cd stress (e.g., 40 μM) secretes chelating metabolites such as citrate and aconitate to reduce Cd^2+^ bioavailability. In contrast, under lower stress levels, it favors metabolites that promote immobilization. Alternatively, in wheat, nucleotides and their degradation products, including extracellular DNA (exDNA), can bind with root mucilage to form a protective anionic barrier. This barrier effectively traps and immobilizes Cd ions, thereby reducing their uptake into the roots [46,47]. Cadmium exposure triggers time-dependent adaptive responses in plants. Under short-term stress (hours to 7 days), plants such as broomcorn millet rapidly secrete phenolic acids (e.g., ferulic acid, coumaric acid), likely to mitigate oxidative damage by activating the phenylpropanoid metabolism. In contrast, long-term exposure (>6 weeks) induces structural adaptations; for example, maize reduces polysaccharide secretion while increasing the lipid-to-protein ratio in root exudates, thereby enhancing cell membrane stability and metal chelation capacity [46,48]. A prominent response is the secretion of organic acids, including low-molecular-weight organic acids (LMWOAs; e.g., malate, citrate) and phenolic acids (e.g., salicylic acid). This process involves specific molecular regulators, such as the FRD3 gene in *Arabidopsis thaliana*, which specifically mediates citrate efflux [49]. For instance, under combined copper-nickel stress, a significant increase in malate secretion has been documented in plants [50]. Similarly, cadmium-exposed mangrove plants exhibited a 2-to 3-fold increase in the secretion of acetic and malonic acids [51]. The detoxification of heavy metals by these organic acids is primarily mediated through three classes of mechanisms. The first involves direct immobilization via complexation and precipitation, such as citrate complexing Cu^2+^ and oxalate precipitating Pb^2+^ [52]. The second mechanism involves the acidification of the rhizosphere environment (e.g., via acetic acid secretion), which alters the solubility and phytoavailability of heavy metals [53]. (3) The third is to maintain the TCA cycle, which ensures a steady supply of carbon skeletons and energy for the continued biosynthesis of organic acids [54]. The fourth mechanism is non-enzymatic ROS scavenging by specific organic acids, such as phenolic acids, which directly neutralize reactive oxygen species to mitigate oxidative stress [55].

Amino acid and carbohydrate secretions exhibit distinct stress-response strategies. Under heavy metal stress, plants typically increase amino acid secretion, as exemplified by a 2- to 3-fold elevation in glutamine secretion in cadmium-exposed rice (*Oryza sativa*) [56]. These amino acids can chelate heavy metals through their ionizable functional groups [57]. Conversely, the secretion of carbohydrates serves primarily to shape the rhizosphere microbiome. For instance, *Leersia hexandra* secretes saccharides (e.g., sucrose) into the rhizosphere, providing a carbon source that enriches specific glucose-metabolizing bacterial taxa. Under high Cd^2+^ exposure, the relative abundances of *Pseudomonadota* and *Bacteroidota* increase to 38.35% and 12.19%, respectively, facilitating a beneficial plant-microbe partnership [50]. Moreover, key metabolic pathways—including carbohydrate metabolism and ABC transporters—were activated. Under heavy metal stress, white clover (*Trifolium repens*) enhances sucrose secretion into the rhizosphere, thereby promoting the proliferation of *Pseudomonas* spp., which mitigates toxicity through siderophore production. These bacterial siderophores chelate Cd^2+^ ions, thereby effectively reducing their bioavailability [58]. Secondary metabolites, including flavonoids (e.g., quercetin), other phenolic compounds (e.g., caffeic acid), and terpenoids, enhance plant resistance through mechanisms such as metal chelation, ROS scavenging, and regulation of ion channels. In response to cadmium stress, Japonica rice (*Oryza sativa* subsp. *japonica*) root-secreted quercetin functions to chelate rhizospheric Cd ions, thereby reducing their bioavailability [56]. Under aluminum stress, the roots of aluminum-tolerant maize (*Zea mays*) profusely secrete phenolic compounds, including caffeic acid. The phenolic hydroxyl groups of these compounds can form stable complexes with Al^3+^ ions in the rhizosphere, thereby preventing their uptake into the root system [59]. Under copper stress, *Elsholtzia haichowensis* roots secrete terpenoids that mitigate Cu toxicity through multiple mechanisms. These compounds reduce Cu^2+^ influx across the membrane by modulating the activity of ion transporters, while also scavenging ROS due to their inherent antioxidant properties. Concurrently, they contribute to membrane stabilization, collectively alleviating copper-induced damage [60].

#### 2.2.2. Impact of Polycyclic Aromatic Hydrocarbons Stress on Root Exudates

Polycyclic aromatic hydrocarbons (PAHs), a class of typically non-polar, hydrophobic aromatic compounds, are ubiquitous persistent organic pollutants (POPs) in various environments [61]. Exposure to PAHs significantly inhibits plant growth and development, manifesting as reduced biomass, shorter plant height, and impaired root function. For example, in Betula platyphylla, shoot and root growth can be reduced by up to 85%, with genotypic variations in sensitivity [62]. Similarly, the root activity of *Salix* spp. Cv. ‘Suliu-172′ declines progressively as phenanthrene concentration increases [63]. Photosynthesis is also a key target of PAH toxicity, often indicated by reduced chlorophyll content and chlorophyll fluorescence parameters, as observed in tomato plants under phenanthrene stress [64]. Exposure to PAHs disrupts the antioxidant defense system, thereby exacerbating oxidative stress. This is directly evidenced by elevated malondialdehyde (MDA) levels, as observed in PAH-stressed tomato plants [64]. The response of antioxidant enzymes often follows a biphasic pattern: low PAH concentrations may transiently stimulate activities of enzymes like POD and SOD, as reported in *Arabidopsis thaliana* [65], whereas high concentrations typically lead to their inhibition due to direct toxicity or severe redox imbalance.

In response to PAH stress, plants activate multi-layered adaptive mechanisms by dynamically modulating the composition and functionality of root exudates [66]. For instance, low-molecular-weight organic acids (LMWOAs), such as oxalic, citric, and tartaric acids, often exhibit a pronounced increase in secretion during the early stages of exposure. Following short-term (7-day) exposure to PAHs, the mangrove species *Aegiceras corniculatum* and *Kandelia* obovate exhibited significantly elevated root secretion of total organic acids, with oxalic acid showing particularly marked increases. Oxalic acid accounted for over 50% of the total root exudates, highlighting its pivotal role in early-stage adaptation to PAH stress [67]. Under phenanthrene and pyrene stress, maize and soybean root exudates showed elevated levels of citrate and succinate. These organic acids enhance the bioavailability of PAHs by improving their solubility and desorption from soil matrices [68]. Conversely, prolonged exposure to PAHs suppresses the secretion of organic acids. For instance, roots of *Bruguiera gymnorrhiza* exhibited reduced secretion of tartrate and malate, declining to levels comparable to controls, which is attributed to PAH-induced suppression of root metabolic activity [69].

PAH stress also significantly alters the secretion profiles of amino acids and sugars. Under combined phenanthrene and pyrene stress, the composition of amino acids in Lolium perenne root exudates changed significantly, with the relative levels of serine, glutamate, and proline increasing by 35%, 28%, and 42%, respectively. These nitrogen-rich metabolites can serve as nutrients for rhizosphere microbiota [70]. Similarly, under benzo[a]pyrene exposure, the secretion of fructose and glucose by *Zea mays* roots increased by 1.8-fold and 2.3-fold, respectively [68]. Under pyrene stress, *Sorghum bicolor* roots exhibited increased secretion of sugars (e.g., glucose), which facilitated biofilm formation by *Pseudomonas* spp. and other pollutant-degrading bacteria. This activation of the microbial quorum-sensing system (AHL signaling pathway) appears to enhance the microbial uptake and utilization of PAHs. Consequently, the co-metabolic degradation capacity of PAHs by rhizobacteria increased by over 30% compared to controls [71]. Under benzo[a]pyrene stress, *Bruguiera gymnorrhiza* exhibited a twofold increase in the secretion of secondary metabolites, particularly phenolic compounds such as 2,4-di-tert-butylphenol. These metabolites contribute to the detoxification of PAHs through redox reactions [69]. Additionally, root exudates of *Medicago sativa* L. exhibited a significant increase in total phenolic content under phenanthrene-contaminated conditions. Correspondingly, flavonoids, including flavanones such as naringenin, increased under these contamination conditions [72].

#### 2.2.3. Impact of HMs and PAHs on Root Exudates

Plant responses to combined heavy metal (HM) and polycyclic aromatic hydrocarbon (PAH) stress are substantially more complex and less predictable than responses to individual stressors. Soil-borne HMs and PAHs are taken up by plant roots and exert synergistic or antagonistic toxic effects. This concurrent stress consequently represses plant growth, development, and photosynthetic processes [73]. Heavy metals primarily disrupt phytohormone signaling (e.g., auxin pathways) and inhibit cell division, resulting in shortened primary roots, thinner lateral roots, and reduced biomass [74,75]. In contrast, PAHs tend to disrupt the lipid architecture of root membranes, impairing water and nutrient uptake. Aboveground, these stresses collectively manifest as stem dwarfism, suppressed leaf expansion, and delayed reproduction [76]. Xu et al. conducted a pot experiment to examine the root system responses of two *Zea mays* genotypes to soil co-contamination with Cd-pyrene during early growth. The results revealed that combined Cd–pyrene pollution significantly reduced stem, leaf, and root biomass in both white and black maize genotypes, with root biomass showing the most pronounced reduction. Furthermore, root surface area, average diameter, and volume in both maize genotypes decreased under Cd–pyrene co-contamination compared to uncontaminated controls [77]. Under combined Cd–benzo[a]pyrene (Cd–BaP) exposure, wheat exhibited a significant reduction in chlorophyll content, including a 16% decrease in the chlorophyll a/b ratio [78]. Furthermore, multiple studies have demonstrated that combined Cd–PAHs exposure suppresses photosynthetic efficiency by reducing Rubisco activity, ultimately compromising plant growth and yield [79].

Plants activate a range of defense mechanisms to mitigate combined HM–PAH stress; however, these responses vary depending on species and developmental stage. This synergistic stress significantly alters the composition of root exudates, as evidenced by elevated levels of water-soluble organic acids (citrate, succinate, and glutarate) in *Scirpus triqueter* under pyrene–lead co-exposure. Notably, citrate secretion increased by 57% at 50 mg/L compared to controls [80].Under combined Cd-pyrene co-stress (20 mg/kg Cd + 30 mg/kg pyrene), *Lolium perenne* roots exhibited a 35–60% increase in malate production and a 25–40% increase in tartrate production, respectively, compared to controls [81].

The metabolic profile of plants, including the pools of compounds available for secretion, is reshaped under combined Cd–pyrene stress. For instance, in *Lolium perenne*, the content of cysteine (Cys)—a key precursor for glutathione and phytochelatins—increased by 25%, enhancing intracellular metal chelation and antioxidant capacity [81]. Concurrently, alterations in amino acid metabolism are observed; in *Festuca arundinacea*, the increased pool of glutamate (Glu), the primary precursor for proline (Pro) biosynthesis, likely supports the synthesis of Pro as an osmoprotectant in response to oxidative stress [82]. Under combined stress, plants accumulate proline (Pro) intracellularly to mitigate osmotic and oxidative stress. This intensive Pro biosynthesis consumes glutamate (Glu) pools, thereby perturbing amino acid homeostasis. This internal metabolic reprogramming is reflected in the composition of root exudates. For example, saccharide secretion often exhibits a concentration-dependent biphasic response: sucrose and fructose levels in exudates may increase under mild stress but decline sharply (by 40–55%) under severe stress, likely due to the reallocation of carbon resources towards internal defense mechanisms and impaired metabolic function [80,83]. Metabolomic analyses have revealed the significant upregulation of key genes in the phenylpropanoid pathway (PAL, C4H). This activation promotes the synthesis of a broad spectrum of secondary metabolites, including flavonoids (which act as antioxidants) and lignin (which reinforces cell wall integrity), collectively enhancing the plant’s physical and chemical barrier against pollutant intrusion [83].This metabolic reprogramming exemplifies the dynamic balance between energy allocation and stress resistance in plants.

Plant root exudate composition and functionality exhibit both shared and specific responses to individual and combined HM and PAH stresses. A common response to both stressors is the frequently observed enhancement in the secretion of low-molecular-weight organic acids (LMWOAs, e.g., citrate, oxalate), which can chelate HMs or solubilize PAHs. Amino acids (e.g., glutamine, proline) and sugars (e.g., sucrose, fructose) are also often secreted, which can indirectly alleviate toxicity by shaping rhizosphere microbiomes, for instance, through the enrichment of *Proteobacteria*. At the level of plant metabolism, HMs predominantly stimulate the synthesis of metal-chelating compounds (e.g., phytochelatins intracellularly, and specific flavonoids like quercetin and metal-complexing organic acids in the rhizosphere), whereas PAHs often promote the production of lipophilic secondary metabolites (e.g., mangrove-derived 2,4-di-tert-butylphenol) that may facilitate PAH oxidative degradation. Under combined stress conditions, HM-PAH interactions can exacerbate the dysregulation of root exudates via synergistic toxicity. It has been proposed that PAHs may increase membrane permeability, potentially accelerating HM influx, while HMs can inhibit the activity of PAH-degrading enzymes, such as laccase. This often triggers a marked increase in the secretion of organic acids (e.g., a 57% increase in citrate in *Scirpus triqueter*) and osmoprotectants like proline and glutathione. These physiological responses are often accompanied by the activation of the phenylpropanoid pathway, which reinforces the cell wall barrier to limit pollutant penetration. There are significant differences in the composition changes in root secretions among different plants under single or combined stress. Table 1 summarizes these changes and their core physiological and ecological functions.

### 2.3. Impacts of HMs-PAHs Stress on Rhizosphere Microorganisms

#### 2.3.1. Impacts of Heavy Metal Stress on Rhizosphere Microorganisms

Heavy metals (HMs), as widespread environmental contaminants, profoundly disrupt the structure and function of rhizosphere microbiomes. The rhizosphere serves as a critical plant-microbe interface for mitigating HM stress. Within this zone, specific microorganisms can enhance metal detoxification, improve nutrient acquisition, and bolster ecological resilience through dynamic interactions with plant roots [84]. Elevated concentrations of heavy metals (e.g., cadmium, lead, copper) in soil trigger changes in root exudate composition, leading to increased release of organic acids, phenolic compounds, and signaling molecules. These altered exudates, in turn, serve as key carbon sources and metabolic substrates, influencing the rhizosphere microbial community. This creates selective pressure that enriches for metal-tolerant microbial taxa and shifts community structure, facilitating plant-microbe interactions that can improve detoxification under sustained HM stress [85]. Heavy metals generally inhibit microbial activity by disrupting cell membranes, suppressing enzymes, and damaging DNA. This often leads to reduced bacterial diversity in the rhizosphere and selects for the proliferation of metal-resistant taxa. Under HM stress, phyla such as *Proteobacteria*, *Bacteroidetes*, and *Verrucomicrobia* often become dominant, while the relative abundance of *Actinobacteria* and *Acidobacteria* may decrease [86]. In specific plant systems, such as hyperaccumulators, this selection is particularly evident. For example, *Sphingomonas* and *Burkholderiaceae* (*Proteobacteria*) form the core microbiota in the rhizosphere of the cadmium hyperaccumulator *Sedum alfredii*, and their abundance is positively correlated with the plant’s cadmium accumulation capacity [86]. In the mangrove rhizosphere, copper stress significantly increased the abundance of copper resistance genes, such as the Cu^+^ efflux pump genes *cusA* and *copA*. Culture-dependent studies from such environments have successfully isolated Cu-tolerant bacteria, including *Vibrio fluvialis* and *Brevibacillus* spp. [87]. Separately, various bacterial strains are known to assist plants under HM stress through different mechanisms. For instance, some Pseudomonas species promote plant growth by secreting phosphatases, while others can reduce toxic Cr(VI) to less mobile Cr(III) through enzymatic pathways [85]. Under heavy metal stress, arbuscular mycorrhizal fungi (AMF), such as *Glomus mosseae*, become significantly enriched in the rhizosphere. Their hyphal networks expand the root absorptive surface area, thereby enhancing phosphorus acquisition efficiency. Furthermore, these fungi immobilize heavy metals within their hyphae and reduce their translocation to plant shoots [88]. Tolerant fungi, such as *Penicillium* spp. and rhizosphere fungi associated with *Arabidopsis thaliana*, secrete organic acids, such as citric acid and oxalic acid, which chelate soluble heavy metal ions, thereby reducing their bioavailability [89]. Furthermore, studies have reported an increased abundance of *Ascomycota* and *Basidiomycota* fungi under prolonged heavy metal stress, which may be attributed to their capacities for metal tolerance and biotransformation [89]. In summary, heavy metal stress typically reduces the overall diversity of rhizosphere microbial communities while selecting for tolerant taxa. These tolerant microorganisms, including specific strains of *Pseudomonas*, *Bacillus*, and arbuscular mycorrhizal fungi (AMF), enhance plant resilience through various mechanisms such as organic acid secretion, metal immobilization, and the activation of resistance genes, collectively driving the restructuring of the rhizosphere microbiome.

#### 2.3.2. Impacts of Polycyclic Aromatic Hydrocarbons Stress on Rhizosphere Microorganisms

Elevated soil concentrations of polycyclic aromatic hydrocarbons (PAHs), such as phenanthrene, pyrene, and benzo[a]pyrene, lead to alterations in root exudate composition, resulting in increased secretion of organic acids, phenolics, and sugars. These compounds serve as essential carbon sources and metabolic substrates for PAH-degrading microorganisms. The altered rhizochemical environment selectively enriches microbial taxa possessing PAH-catabolic genes, thereby driving the adaptive restructuring of the community towards a more PAH-degrading profile [90]. PAH stress typically reduces rhizosphere bacterial diversity while increasing the relative abundance of stress-tolerant and PAH-degrading taxa. This often results in the marked enrichment of specific bacterial groups, such as certain lineages of *Proteobacteria* and the genus *Sphingomonas*. For example, in PAH-contaminated alpine soils, *Proteobacteria* were reported to comprise 35.3% to 98.45% of the bacterial communities [91]. Under PAH stress, rhizosphere microorganisms with intrinsic or horizontally acquired PAH-catabolic genes (e.g., alkB, PAH-RHDα), such as *Pseudomonas*, *Sphingomonas*, *Mycobacterium*, and *Rhodococcus*, are often enriched. The degradation efficiency of PAHs by these microbes can be further regulated through quorum-sensing mechanisms [90]. For instance, the *nahH*-encoded catechol 2,3-dioxygenase in *Pseudomonas* spp. catalyzes the conversion of phenanthrene into catechol intermediates, which are subsequently mineralized into CO_2_ and water. Long-term PAH stress often shifts fungal community composition, promoting the dominance of *Ascomycota* and *Basidiomycota*, as revealed by metagenomic analyses of chronically contaminated soils [91]. Under prolonged exposure, fungal taxa such as *Aspergillus* and *Penicillium* spp. can degrade PAHs, including high-molecular-weight compounds like pyrene and benzo[a]pyrene, through the secretion of non-specific enzymes such as lignin peroxidase (LiP) and laccase. Arbuscular mycorrhizal fungi (AMF), such as *Rhizophagus irregularis*, may also interact with PAHs. Their extensive hyphal networks can influence the rhizosphere microenvironment and plant health, while the fungal cell walls (composed of chitin and melanin) can sorb PAHs, potentially reducing their immediate bioavailability [92].

#### 2.3.3. Effects of HMs-PAHs Combined Stress on Rhizosphere Microorganisms: An Integrated Analysis

The interactions between heavy metals (HMs) and polycyclic aromatic hydrocarbons (PAHs) under combined stress significantly alter rhizosphere microbial processes, often leading to synergistic toxicity that exceeds the impact of individual pollutants. The specific responses of microbial taxa and their synergistic functions with plants under single and combined stresses are summarized in Table 2. A key interaction is the inhibition of PAH biodegradation by HMs, potentially through metal toxicity to degrading microbes. Concurrently, PAHs can exacerbate stress by impairing overall microbial metabolism, which may indirectly reduce the community’s capacity for heavy metal immobilization. The response of specific microbial taxa to combined HM-PAH stress can vary depending on the specific contaminants, plant species, and soil conditions. For example, in the rhizosphere of *Carmona microphylla* under Cd-PAH stress, *Proteobacteria* and *Bacteroidetes* became dominant while *Actinobacteria* declined [93]. In contrast, a study on *Lolium perenne* under Cr-PAH stress reported an increase in the abundance of *Actinobacteria*, *Proteobacteri*a, and *Bacteroidetes*. This suggests that the response of *Actinobacteria* is context-dependent. Despite these variations in community composition, beneficial bacteria such as *Pseudomonas* are often enriched and are known to assist plant adaptation under stress, for instance, through the secretion of organic acids [81]. Additionally, arbuscular mycorrhizal fungi (AMF), such as Glomus mosseae, can immobilize heavy metals within their extensive mycelial networks, reducing their translocation to plant shoots. Under combined Cd–PAH stress, fungi such as *Penicillium* species (Ascomycota) can secrete organic acids (e.g., citric acid) that chelate cadmium, thereby reducing its bioavailability [93,94]. Beyond taxonomic shifts, microbial interactions also change. Fungal co-occurrence networks often exhibit greater structural stability and niche adaptability compared to bacterial networks under stress. The impact of HMs on fungi can be dose-dependent, with low concentrations sometimes stimulating growth and high concentrations being inhibitory. Furthermore, the assembly of fungal and bacterial communities may follow different rules; for instance, under PAH stress, fungal communities may be more influenced by stochastic processes (e.g., dispersal limitation), while bacterial communities are more shaped by deterministic selection [84].

In summary, exposure to HMs and PAHs, whether single or combined, typically reduces rhizosphere microbial diversity and enriches for pollutant-tolerant taxa such as *Proteobacteria* (e.g., *Pseudomonas*) and fungi like *Penicillium*. These microorganisms mitigate pollution stress by activating specific resistance genes (e.g., metal efflux *cusA*, *copA*; PAH degradation *alkB*, *PAH-RHD*) and secreting metabolites (e.g., organic acids, extracellular enzymes). The response of specific groups varies: *Actinobacteria* are often inhibited by HMs, while PAH stress enriches degraders like *Mycobacteria* and ligninolytic *Basidiomycota*. Under combined stress, the antagonistic interactions between pollutants can exacerbate toxicity, placing a greater burden on these tolerant microbial taxa to maintain rhizosphere function and assist in phytoremediation.

### 2.4. Synergistic Interactions Between Root Exudates and Rhizosphere Microbes for Alleviating HMs-PAHs Stress

#### 2.4.1. Repair Efficiency and Mechanism of Heavy Metals by Root Exudates and Rhizosphere Microorganisms

Plant root exudates contribute to heavy metal remediation through several key mechanisms. Firstly, plants can modulate the pH of the rhizosphere through proton (H^+^) or bicarbonate (HCO_3_^−^) exudation. The specific response is metal- and plant-dependent. For instance, some plants acidify the rhizosphere to enhance the phytostabilization of certain metals, while for others, an increase in pH can promote metal precipitation and reduce bioavailability [96]. Secondly, plants can release reactive oxygen species (ROS), such as hydrogen peroxide (H_2_O_2_), into the rhizosphere, which can oxidize certain heavy metals (e.g., As, Cr) and alter their solubility and toxicity [97]. Furthermore, organic acids in root exudates play complex roles. They can form stable water-soluble complexes with metal ions, increasing their mobility and potential for leaching or plant uptake. Conversely, under different conditions, these metal–organic acid complexes can facilitate the precipitation of metals or reduce their direct phytotoxicity by chelation, thereby reducing plant exposure [98,99]. Furthermore, root exudates serve as carbon and energy substrates for rhizosphere microorganisms, thereby stimulating their growth and metabolic activity. This enhanced microbial activity indirectly modulates heavy metal bioavailability, thereby facilitating plant–microbe partnerships in remediation.

Root exudates and microorganisms can synergistically enhance the phytoremediation of heavy metal pollution. For example, inoculation with the endophytic fungus *Piriformospora indica* significantly increased plant uptake of Cd and Cr by 31.5–88.9% and 22.4–38.4%, respectively. This was linked to fungal-induced plant growth promotion, changes in root exudate profiles (e.g., lipids and organic acids), and restructuring of the rhizosphere microbiome. Associated with these changes, a decrease in soil heavy metal concentrations (or an increase in removal rates) was also observed [100]. In *Miscanthus* × *giganteus*, root exudates (comprising 26.74% lipids and phenols) were associated with an altered microbial community and enhanced formation of larger soil aggregates (a 9.78% increase compared to control). Within these aggregates (2–0.25 mm fraction), Pb was enriched by 15.3%, and Cd bioavailability decreased by 28%, suggesting a potential mechanism for metal immobilization in the rhizosphere [101].

#### 2.4.2. Repair Efficiency and Mechanism of Polycyclic Aromatic Hydrocarbons Stress by Root Exudates and Rhizosphere Microorganisms

Root exudates critically enhance the phytoremediation efficiency of PAHs under stress conditions through multipathway interactions. While plant roots secrete various enzymes (e.g., invertase, phosphatase, protease) into the rhizosphere, these primarily act on simple substrates and contribute to general nutrient cycling. The direct enzymatic degradation of complex PAHs is predominantly carried out by microbial oxidoreductases (e.g., dioxygenases, laccases, peroxidases), whose activity and production can be stimulated by root exudates [79]. Notably, certain plant-derived peroxidases can catalyze the oxidative polymerization of phenolic compounds and some aromatic contaminants, leading to their incorporation into soil organic matter and reducing their immediate bioavailability [102]. More importantly, the primary mechanism for PAH degradation is the stimulation of pollutant-degrading microorganisms by root exudates. Empirical studies confirm that microbial populations and metabolic activity in the rhizosphere are significantly higher than in bulk soil, which accelerates the microbial oxidation and mineralization of PAHs, thereby enhancing biogeochemical cycling [103].

The synergy between root exudates and microorganisms in PAH degradation has been demonstrated across diverse plant systems. In woody plants, mangrove species (*Avicennia* spp.) secrete low-molecular-weight organic acids and amides, which are associated with high removal rates (87–97%) of phenanthrene, pyrene, and benzo[a]pyrene within 45 days. A significant negative correlation between the secretion levels of these compounds and residual PAH concentrations suggests a potential functional role in reducing contaminant persistence [104]. In herbaceous plants, ryegrass and rice enrich for degraders like *Acinetobacter* spp., achieving up to 97% benzo[a]pyrene degradation in 40–50 days. Root exudates here are thought to reduce the half-life of benzo[a]pyrene by improving its bioavailability [105,106]. Similarly, exudates from maize and soybean enhanced PAH removal in aged soils by 69.2–78.4% [68]. Beyond providing carbon sources, some exudates directly activate degradative enzymes. For example, caffeic acid from sorghum roots can synergistically enhance peroxidase-mediated oxidation of phenanthrene [107]. Microbial cooperation is pivotal. Consortia of *Rhodococcus* and *Burkholderia* secrete dioxygenases and laccases, achieving up to 99% pyrene degradation [90]. Mycorrhizal fungi like *Glomus mosseae* enhance PAH bioavailability by secreting biosurfactants such as rhamnolipids, significantly reducing PAH concentrations in the rhizosphere [108]. The principles of this synergy can be applied in engineered remediation. For instance, the application of simulated root exudates (e.g., sodium linoleate) achieved 40.6% PAH removal, while composite agents combining biochar with specific bacteria showed removal rates 20–40% higher than bacterial agents alone, by mimicking and optimizing the natural rhizosphere microenvironment [109].

#### 2.4.3. Remediation Efficiency and Mechanism of Root Exudates for HMs-PAHs Contaminated Soils

Phytoremediation mechanisms under combined heavy metal-polycyclic aromatic hydrocarbon (PAH) co-contamination are more complex than under single-pollutant stress. Pollutant interactions can bidirectionally alter remediation efficiency: they can be synergistic, enhancing removal, or antagonistic, inhibiting it. For example, copper co-contamination was shown to enhance alfalfa’s remediation of benzo[a]pyrene by stimulating plant growth and microbial activity [110]. Similarly, low lead concentrations can synergistically increase benzo[a]pyrene uptake in ryegrass, whereas high lead levels inhibit remediation due to toxicity [111]. Conversely, other studies have documented antagonistic effects where the presence of one pollutant significantly reduces the removal of the other.

The interaction between root exudates and rhizosphere microorganisms is considered a potential key driver of phytoremediation under combined pollution stress, although research in HM-PAH co-contaminated systems remains limited and the outcomes are context-dependent. The proposed mechanisms involve a complex interplay: root exudates (e.g., organic acids, enzymes, sugars) can shape the microbial community by serving as carbon substrates and by modifying the rhizosphere environment (e.g., through acidification). These changes can concurrently influence the fate of both HMs and PAHs; for instance, organic acids may chelate and immobilize HMs while simultaneously enhancing the bioavailability of PAHs for microbial degradation. The enriched microorganisms, in turn, may contribute to remediation through various mechanisms, including biofilm formation, redox transformations, secretion of metabolites (e.g., siderophores, phosphatases), and arbuscular mycorrhizal symbiosis. A hypothesized framework suggests that heavy metals might exert a stronger selective pressure on microbial community structure due to their direct toxicity, while PAH-induced shifts might be more linked to the availability of specific carbon sources and metabolic intermediates. It is further proposed that the complexity and stability of the root exudate-microbial interaction network could be an important factor influencing the robustness and efficiency of the remediation process in these complex environments.

## 3. Conclusions

**Figure 1:**
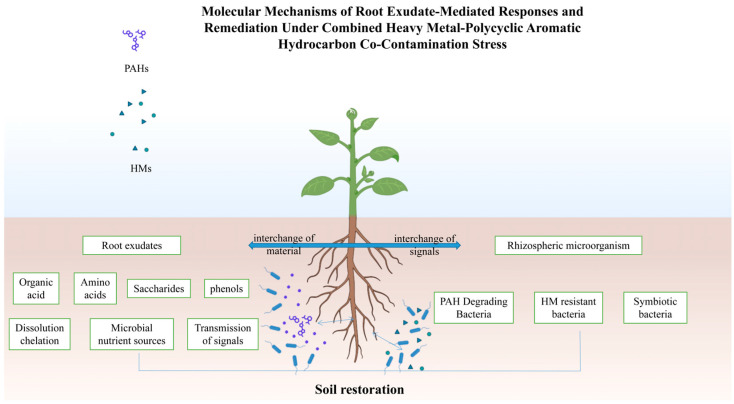
The rhizosphere of plants enhances the removal efficiency of heavy metals and organic pollutants by increasing bioavailability and biodegradation.

This review synthesizes the pivotal role of root exudates in orchestrating the phytoremediation of soils co-contaminated with HMs and PAHs, a process whose core mechanism is visually summarized in Figure 1. Previous studies have demonstrated that under combined stress, plants dynamically adjust the composition of their root secretions, thereby actively shaping a functional rhizosphere microbiota. The resulting plant-microbe partnership establishes a self-amplifying remediation cycle: root exudates enhance contaminant bioavailability and facilitate specific microbial recruitment, while the enriched microbial consortia, in turn, contribute to pollutant degradation and improved plant health.

However, the practical application of this root exudate-mediated mechanism faces significant challenges. Key bottlenecks include the unresolved dynamic regulation of exudate profiles under complex stress, creating a predictive “black box”: the dual effects of the exudate-shaped microenvironment, where metal speciation changes may inadvertently enhance PAH bioavailability; the disruption of chemical signaling under high pollutant loads, leading to microbial recruitment failure; and the formidable gap between laboratory findings and field applications, where beneficial exudation phenotypes often suffer from functional attenuation due to soil adsorption and degradation.

To translate this mechanistic understanding into effective rhizosphere engineering strategies, future research must focus on overcoming these hurdles. Prioritized efforts should include the following: elucidating the key regulatory nodes controlling exudate synthesis to develop plant “training” strategies; screening for and constructing efficient plant-microbe partnerships using degraders or resistant bacteria that utilize target exudates; developing low-cost soil amendments to protect secreted compounds and prolong their activity; and establishing robust in situ monitoring techniques to accurately track exudate dynamics in field conditions. Through these integrated approaches, the full potential of this green, sustainable remediation strategy can be unlocked for the rehabilitation of complexly polluted environments.

## Figures and Tables

**Table 1:** The changes in root secretions under different stresses.

Stress Type	Representative Secretion Changes	Core Physiological and Ecological Functions
HMs (e.g., Cd, Pb, Cu)	increased secretion of low-molecular-weight organic acids (including citrate and malate), and enhanced accumulation/secretion of secondary metabolites (like phenolics and flavonoids).	Key Mechanisms of Detoxification and Immobilization: Reduced metal bioavailability via chelation and precipitation. Activated antioxidant defenses encompassing both enzymatic and non-enzymatic systems to alleviate oxidative stress.
PAHs (e.g., Phe, Pyr, BaP)	Characterized by an early enhancement of low-molecular-weight organic acids (e.g., oxalate, citrate) and a concomitant increase in sugars and amino acids that provided essential carbon and nitrogen sources for soil microbes.	Key Mechanisms of Solubilization and Co-metabolism: Increased PAH bioavailability by promoting their desorption from soil particles. Provided substrates and energy to microbial communities to facilitate co-metabolic degradation.
HMs-PAHs	The secretion of specific organic acids (e.g., citrate, succinate) displayed both synergistic and antagonistic dynamics. This was accompanied by a pronounced upregulation of secondary metabolic pathways and the synthesis of osmolytes such as proline.	Synergistic Resistance and Trade-offs: While the plant mounted a coordinated defense against both contaminants, the interplay between the two stressors could disrupt the root exudate profile, representing a significant physiological trade-off. To maintain cellular integrity under these conditions, the plant reinforced its cell walls (via lignification) and enhanced its oxidative stress defense systems.

**Table 2:** The changes in plant root microbiota under different stress conditions.

Stress Type	Rhizosphere Microorganisms	The Role of Root Exudates in Mediating Interactions and Remediation Mechanisms	Literature Source
HMs(Pb)	*Brevibacterium frigoritolerans* YSP40, *Bacillus paralleleniformis* YSP151	The bacterial strain improved lead (Pb) phytoextraction via two concurrent mechanisms: it acidified the rhizosphere by secreting organic acids, which solubilized refractory Pb minerals (e.g., PbO and PbCO_3_) and increased Pb bioavailability; while simultaneously promoting plant biomass through the secretion of the phytohormone IAA. Ultimately, this dual action elevated Pb accumulation in the mustard plants by up to 4.6-fold.	[81]
PAHs	*Sphingomonas*, *Pseudomonas*, *Bacillus*, *Mycobacterium*, *Rhodococcus*	Root exudates (e.g., organic acids, sugars, amino acids) provide carbon and nitrogen sources that enrich PAH-degrading bacteria. These microbes possess PAH-RHD genes (encoding ring-hydroxylating dioxygenase), which drives the initial hydroxylation and degradation of PAHs. The exudates also enhance PAH bioavailability, collectively promoting contaminant removal.	[90]
HMs-PAHs	*Actinobacteria*, *Proteobacteria* (e.g., *Sphingomonas*, *Pseudomonas*)	In response to the combined stress of HMs and PAHs, ryegrass altered its root exudate profile (e.g., organic acids, amino acids, and phenolics). This shift selectively enriched specific microbial taxa equipped with dual functions for tackling both contaminants. HMs immobilization was primarily mediated by *Actinobacteria*, which secreted organic acids like citrate and oxalate to chelate HMs, effectively reducing its bioavailability. PAH degradation was driven by genera such as *Sphingomonas* and *Pseudomonas*. These bacteria utilized root-derived carbon to express the PAH-RHDα gene, catalyzing the initial hydroxylation and ring-cleavage of PAHs.	[95]

## Data Availability

The raw data supporting the conclusions of this article will be made available by the authors on request.
